# Bee‐Mediated Pollen Transport Across Five Urban Landscape Features: Buildings Are Important Barriers

**DOI:** 10.1002/ece3.71339

**Published:** 2025-04-24

**Authors:** Olivér I. Roper, Elsa Youngsteadt

**Affiliations:** ^1^ Department of Applied Ecology North Carolina State University Raleigh North Carolina USA; ^2^ Department of Biological Sciences North Carolina State University Raleigh North Carolina USA; ^3^ Center for Geospatial Analytics North Carolina State University Raleigh North Carolina USA

**Keywords:** connectivity, fluorescent dye, pollen dispersal, pollinator ecology, urban bees, urban ecology

## Abstract

Urbanization alters insect pollinator diversity and foraging ranges, while also providing novel pollinator habitats. Common urban landscape features, such as roads and buildings, may alter the ability of insect pollinators to move and forage throughout the urban landscape. In this study, we aimed to quantify the effects of common urban landscape features on insect pollinator movement. We focused on roads, buildings, forest fragments, lawns, and community gardens. We studied five community garden sites and the landscape features surrounding them in Raleigh, North Carolina, USA. To measure pollinator movement across each feature, we placed clusters of potted cucumber plants on either side of a feature and added fluorescent dye powder to the stamens of the flowers. After 7 h, we collected and counted the number of fluorescent dye powder grains transferred to each cucumber stigma. We conducted 10‐min visitation observations at each cluster to assess the pollinator community and to assess whether low visitation was linked to low dye transfer. Buildings had the lowest estimated dye transfer, roads and gardens were intermediate, and lawns and forest fragments had the highest estimated dye transfer. Although plants associated with buildings also had low visitation rates, visitation overall was a poor predictor of dye transfer. The most common visitors observed were *Apis mellifera* , *Bombus* spp., and *Xylocopa virginica* , indicating our results are likely primarily representative of these large, generalist bee species. Our study highlights the heterogeneity of urban spaces to pollinators. We demonstrate which features facilitate and inhibit movements of pollinators and thereby provide an empirical basis to map and assess functional landscape connectivity. This information can help cities identify and create connected networks of habitat for essential pollinators using geospatial methods, and can inform research about resource accessibility and foraging energetics for urban pollinators.

## Graphical abstract

Urban landscape features may disrupt or facilitate the foraging movements of insect pollinators; for example, bees are more likely to move along roads than across them. Here, we investigated not only roads but also lawns, gardens, forests, and buildings by measuring the transfer of fluorescent dye between flowering plants separated by each feature. Buildings were the least likely to be crossed, while lawns and forests were the most likely to be crossed, suggesting that fine‐scale urban landscape composition can shape pollinator access to resources and plant mating opportunities.

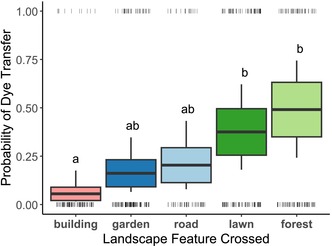

## 1 Introduction

Across the globe, urbanization is projected to increase by 40%–67% between 2013 and 2050 (Li et al. 2019). In certain countries, the United States, for example, this growth includes urban sprawl (also named low density urban land cover growth) (Angel et al. 2011; Terando et al. 2014). These sprawling urban landscapes tend to modify resource use, alter gene flow, and restrict wildlife movements within the confines of habitat patches (Jha and Kremen 2013; Larson et al. 2020; Miles et al. 2019; Parsons et al. 2019). However, much of urban wildlife movement research is conducted on vertebrates (e.g., GPS collars and easier individual tracking), leaving urban invertebrate movements largely understudied (e.g., Doherty et al. 2021). Insects, however, represent the vast majority of animal species on the planet (Mora et al. 2011; Stork 2018) and perform important urban ecosystem functions such as nutrient cycling and pollination (Kotze et al. 2022). Further, insect‐pollinated urban plants must rely on pollinators for pollen dispersal. Accordingly, pollen movement is related to insect movement and barriers to pollinator movement may also restrict pollen dispersal and plant gene flow (Youngsteadt and Keighron 2023). Given growing interest in urban pollinator conservation (Braman and Griffin 2022), as well as the role of insect movements in urban pollen dispersal (e.g., Van Rossum 2010), a better understanding of urban pollinator movement is needed.

Urbanization has complex effects on insect communities, generally reducing diversity and altering species compositions compared to natural or semi‐natural areas (Ahrné et al. 2009; Baxter‐Gilbert et al. 2015; Fenoglio et al. 2020) (Fischer et al. 2016; Geslin et al. 2016; Martin et al. 2018; Wagner 2020). At the same time, urban spaces can provide floral and nesting resources that support insect pollinators compared to other intensive land uses such as agriculture (Samuelson et al. 2022; Theodorou et al. 2020). However, the potential role of urban habitat connectivity in shaping pollinator access to resources and plant mating opportunities is poorly understood. On a large geographic scale, urban areas can be barriers to regional insect gene flow, as was the case in the bumble bee *Bombus vosnesenskii* (Jha and Kremen 2013). On a more local scale, dense cities can change bumble bee foraging ranges and stingless bee homing distances compared to natural or semi‐natural areas (Conflitti et al. 2022; Wayo et al. 2022). These suggestive results do not indicate which specific aspects of an urban landscape alter pollinator movement. Several studies demonstrate that bees and other insects are more likely to move along roads than across them (Askling and Bergman 2003; Dániel‐Ferreira et al. 2022; Fitch and Vaidya 2021; Markovits et al. 2025), thus providing one specific mechanism by which urban areas may limit pollinator movement, gene flow, and access to resources.

To understand urban pollinator movement on a landscape scale, there is a need to evaluate how insects interact with other urban landscape features, not only roads. For example, Johansson et al. (2018) assigned values of floral abundance and landscape friction (a measure of permeability for species) to multiple features such as lawns, gardens, and forests to provide a continuous map of both resources and barriers throughout a focal city. Pollinator abundance and diversity within focal sites depended on landscape scale floral resources surrounding the sites—but only when those resource estimates accounted for barriers. Resources within a fixed, Euclidean distance of sites, ignoring barriers, did not predict pollinator abundance and diversity. This result suggested that urban landscape heterogeneity shapes pollinator access to resources, likely through barriers to foraging movements. The assigned values of landscape friction, however, were based on expert opinion rather than movement data. Studies in vertebrates find that estimates of landscape friction or resistance are most valuable when derived from empirical movement data rather than expert opinion (LaPoint et al. 2013; Zeller et al. 2012).

Accordingly, this study aims to explore how common urban landscape features—roads, buildings, forest patches, lawns, and gardens—affect insect pollinator movement and pollen dispersal in a growing US city, with the goal of gaining data that can be applied to city‐wide landscape friction maps (e.g., Unnithan Kumar et al. 2022) to predict urban pollinator movement and better understand connectivity among resource patches for pollinators within cities. To do so, we quantify the movement of fluorescent powdered dye among cucumber flowers separated by different urban landscape features. To the extent that the probability of dye movement represents insect pollinator movement and pollen dispersal, we interpret features with the lowest probability of dye movement as barriers to insect pollinator movement. Similarly, a high probability of dye movement might indicate features that facilitate insect pollinator movement. We predicted that roads, buildings, and forest fragments would represent significant barriers and that gardens and lawns would facilitate movement.

## 2 Methods

### 2.1 Study Sites and Species

All study sites were centered on community gardens in Raleigh, NC, USA, a city that exemplifies the recent growth and sprawl of urban areas in the biodiverse southeast region of the US (Terando et al. 2014; U.S. Census Bureau 2023). We chose five community garden sites that were a subset of those already included in a larger ongoing pollinator study. These five focal sites were selected based on the presence of five surrounding common urban landscape features, identified using an aerial basemap in ArcGIS Pro. Focal landscape features were roads, buildings, forest fragments, lawns, and the community gardens themselves. Together, roads, buildings, lawns, and trees comprise 92% of the remotely sensed land cover in the city of Raleigh (Zhang et al. 2022), so understanding the effects of these features on pollinator movement will contribute to predicting connectivity on a landscape scale. In our study, roads were two lanes and had speed limits of 45 mph; focal plants were placed on road verges that had mowed grass and sparse wildflowers (similar to those on lawns). Buildings were one storey (mean ± SD area = 1361 m^2^ ± 703 m^2^; width = 36 m ± 17 m; depth = 48 m ± 26 m), and focal plants were placed on lawn or sidewalk on either side of the building. Forest fragments had an average of 70% canopy cover (based on the National Land Cover Database canopy product for 2021 [Housman et al. 2023]) consisting of pine and mixed hardwood species characteristic of the North Carolina Piedmont (e.g., American beech [ *Fagus grandifolia* Ehrh], loblolly pine [ *Pinus taeda* L.], red maple [ *Acer rubrum* L.], and oaks [*Quercus* spp.]) with leaf litter and some invasive species such as Japanese honeysuckle ( *Lonicera japonica* Thunb.); focal plants were placed in lawns adjacent to and separated by the forest fragments. Lawns were regularly mowed and contained sparse native and non‐native wildflowers [e.g., dandelions [*Taraxacum officinalis*], white clover [ *Trifolium repens* L.], woodsorrel [*Oxalis* spp.], and plantains (*Plantago* spp.)] with an average of 14% canopy cover (Housman et al. 2023). Finally, the community gardens ranged from 464 to 4759 m^2^ and grew a mix of ornamental flowers (e.g., scarlet beebalm [ *Monarda didyma* L.], *Dahlia* spp., sunflowers [*Helianthus* spp.], *Aster* spp., coneflowers [*Echinacea* spp.], and marigold [*Tagetes* spp.]) and crop plants (e.g., cucumbers [ *Cucumis sativus* L.], tomatoes [ *Solanum lycopersicum* L.], squash [*Cucurbita* spp.], peppers [ *Capsicum annuum* L.], lavender [ *Lavandula stoechas* L.], and oregano [ *Origanum vulgare* L.]). Focal cucumber plants were placed adjacent to and separated by garden beds. Research permissions for all sites were provided by garden managers, private property owners, or the City of Raleigh Department of Parks, Recreation, and Cultural Resources.

As focal plant species for pollinators to visit, we used cucumber ( *Cucumis sativus* L.), a common garden plant with monoecious, insect‐pollinated flowers that are visited by generalist pollinators, primarily bees (Lowenstein et al. 2012, 2015; Stanghellini et al. 2002). Cucumber was ideal for our study design because it can grow well in containers (Crane et al. 2018), because pollen movement depends entirely on insects (Delaplane 2023), and because its flowers last a single day, ideal for our dye transfer study (below). Cucumbers were Vlaspik F1, a gynoecious variety (88%) mixed with pollenizer variety Sire R (12%) (TS&L Seed Company, Woodland, CA, USA). The plants were grown and stored outdoors in a team member's yard in two‐gallon pots with two plants per pot, trellised on bamboo stakes. The pots were filled with Sunshine Mix #1 (Sungro Horticulture, Agawam, MA, USA) and slow‐release fertilizer pellets at the label rate (Osmocote Smart‐Release Flower & Vegetable, 14‐14‐14, Scotts Miracle‐Gro, Marysville, Ohio) with water provided ad libitum. We grew two batches of cucumbers, one deployed at study sites in June and one in July, 2023.

### 2.2 Data Collection

#### 2.2.1 Study Design

**FIGURE 1:**
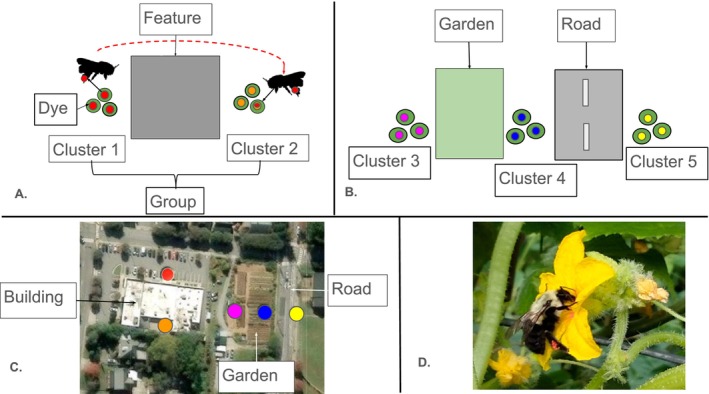
Study design and study system. (A) and (B) Diagrams showing the two setup designs used, where (A) shows a bee transferring fluorescent dye powder from one side of an urban landscape feature (Cluster 1) to the other (Cluster 2). Clusters consist of 3 pots of cucumber plants (green circles), are 20–30 m apart, and are separated by a feature (gray square). A group refers to associated clusters that trade dye. Multicolored dots among clusters in (B) represent different colored dye used in each cluster; when two features were adjacent to one another, a central cluster of plants could transfer dye across both features. The map (C) shows an example of a site where the building clusters represent design (A) and the garden and road clusters represent design (B). Dots in (C) represent clusters shown in both (A) and (B). Panel (D) is a photograph of a *Bombus impatiens* worker visiting a dyed flower. Panel C was created using Esri ArcGIS Pro 3.2.2 software with the World Imagery base map imagery provided by Esri, Maxar, Earthstar Geographics, and the GIS user community.

To assess bee movement across landscape features, we positioned potted cucumber plants in clusters at the five study sites. Here, a “cluster” of plants refers to three pots placed together to create a resource patch attractive to pollinators. Each focal landscape feature received two clusters, separated by the feature, such that dye transfer between clusters would represent pollinator movement across that feature (Figure 1). Across all clusters, we aimed to keep total flower displays similar (mean ± SD flowers per cluster = 37 ± 13 in June and 44 ± 12 in July). Clusters were placed 20–30 m apart (mean ± SD = 24 ± 3 m, Table A1; a single fixed distance was not feasible in our landscapes, but distance was independent of feature type). We deployed the clusters at 8:00 and collected them later the same day at 15:30 to allow for ~7 h of data collection per sampling day. Sampling days were June 26–30 and July 17–21, 2023. Each site was sampled once per round.

#### 2.2.2 Fluorescent Dye Powder Application, Collection, and Counting

To detect bee movement across urban landscape features, we marked flowers with fluorescent dye. Despite growing interest in technologies for tracking individual insects (e.g., Green II 2023; Kim et al. 2019; Kissling et al. 2014), these approaches remain expensive; many are limited to large insects and can take a heavy toll on insect behavior (Batsleer et al. 2020). The use of fluorescent dye as a pollen analog is a well‐documented and accessible method for tracking pollinator movement (Huais et al. 2022; Kearns and Inouye 1993; Van Rossum et al. 2011), including movement across roads (Dániel‐Ferreira et al. 2022; Fitch and Vaidya 2021). Dye movement provides an overall measure of the extent to which a pollinator community moves between different flowers or patches of flowers; it does not indicate which individual insect species are responsible for the dye transfer.

We applied fluorescent dye powder (hereafter dye) to the stamens of cucumber flowers using wooden toothpicks (Kearns and Inouye 1993). Toothpicks were discarded after each use. We used a total of eight colors and randomly assigned one color per cluster within each site‐date combination; thus, no color was consistently associated with a particular plant, location, or landscape feature. This randomization ensured that any insect behavioral responses to specific dye colors would not bias the results. These colors included pigments from DayGlo Color Corp, Cleveland, OH, USA (A‐17‐N Saturn Yellow, A‐18‐N Signal Green, A‐11 Aurora Pink), from Radiant Color, Houthalen, Belgium (blue), and from Rolio Pigments, Simi Valley, CA, USA (pure white, neon orange, deep orange, and light orange). We chose colors based on our ability to distinguish between pigments viewed with a dissecting microscope under UV light. We applied dye as soon as all cucumber clusters were set out in the morning, between 08:30 and 09:30.

At the end of the day (between 14:45 and 17:30), we collected the stigmas of cucumber flowers, moving among clusters in the same order we applied the dye earlier in the day. We used forceps to dissect the female cucumber flowers, cleaning the forceps with 70% ethanol and Kimwipes between each dissection, then collected each stigma into a clean 1.5 mL microcentrifuge tube. These tubes were placed in a freezer for later dye particle counting. After collecting all the stigmas from cucumber clusters, we removed all remaining flowers and rinsed the plants with water; we reused these plants the next sampling day when new flowers opened. We assembled plant clusters de novo on each sampling day to ensure that female flowers were present in each cluster, and that all clusters on a given day had a similar number of flowers. Thus, no individual pot had a consistent association with other plants, a particular dye color, or landscape feature.

**FIGURE A1:**
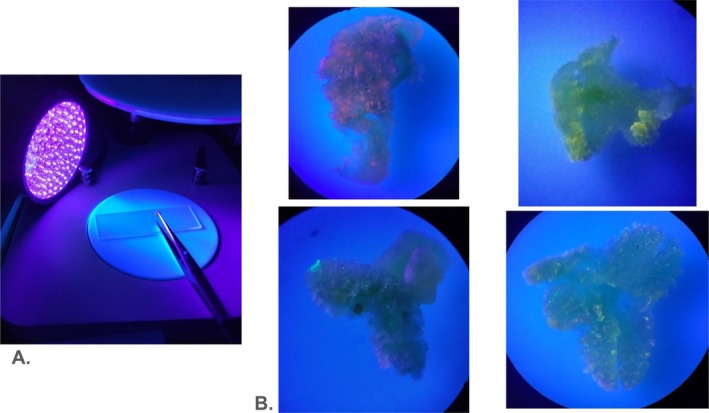
Fluorescent dye particles on cucumber ( *Cucumis sativus* ) stigmas under a dissecting microscope. (A) The setup of our UV lamp and the stigma placed on a microscope slide with tweezers. Both the microscope slide and tweezers were cleaned with 70% ethanol and Kimwipes between each counting. (B) Examples of stigmas under a microscope view with various dye colors that were transferred by bees. Here magnification is set to illustrate the entire stigma, but magnification was increased as needed to count particles.

To quantify dye movement across urban landscape features, we recorded the absence/presence and number of dye particles that were a different color than the one originally applied to a cluster (indicating that dye from another cluster had been transferred by insects). Since we were typically able to add dye to every cluster at a site, we detected movement going in both directions across a feature. Dye particle counting was conducted blind to treatment to reduce observer bias. Stigmas were cut in half using a razor and laid flat so both sides were visible. We used a UV flashlight (UVBe‐100, uvBeast, Portland, OR, USA) and dissecting microscope (45x) in a dark room to view and count dye particles on stigmas (Figure A1).

#### 2.2.3 Insect Visitation Observations

To document the insect community visiting cucumber flowers during our sampling days, we performed insect observations for 10 min at each cluster on each sampling day. Observations were held from ~10:00 to 11:00 after applying dye to each cluster, again, going in the same order the dye was applied in. Observers were trained entomologists familiar with local pollinator species and specimen‐based collections previously taken from cucumber flowers at the same sites (Table A2). Each observer sat at each cluster and recorded every time a visitor landed on a flower. Each separate landing on a flower, including by the same individual insect, counted as a visit. An individual only landing on the flower, but not collecting pollen or nectar, was not counted, since such visits could not result in pollination or dye transfer. Bees were identified on the wing to genus or species, and other insects to genus, family, or order. No specimens were collected because we did not want to disturb insect foraging and movement behavior throughout the 7‐h dye transfer period.

### 2.3 Data Analysis

All data analyses were performed in R 4.4.1 and RStudio 2024.06.10 (Posit Team 2023; R Core Team 2023). For all analyses, we fit generalized linear mixed models using the package ‘lme4’ (Bates et al. 2015), then checked residual diagnostics with ‘DHARMa’ (Hartig 2022), and tested model significance with a Wald *X* ^2^ test using “car” (Fox and Weisberg 2019). For post hoc comparisons among landscape features, we made all pairwise comparisons using the Tukey method in “emmeans” (Lenth 2023).

#### 2.3.1 Fluorescent Dye Movement

To assess dye movement across urban landscape features, we modeled the absence/presence of dye particles per stigma as a function of the urban landscape feature that separated that stigma from the dye source cluster. To account for differences between sites, weather conditions, and cluster placements within sites, we included random intercepts of plant cluster nested in plant group nested in date nested in site. (Here, “group” refers to the 2–3 nearby clusters that could have traded dye across focal landscape features, Figure 1). The model was fit with binomial distribution and logit link. We also performed these tests for dye particle counts in a model with the same structure, fit with Poisson distribution and log link, including an observation‐level random intercept to correct overdispersion in the residuals.

#### 2.3.2 Insect Visitation

Low levels of dye movement across a landscape feature could arise from low visitation rates to either plant cluster associated with that feature, or from high visitation rates from insects that did not cross the feature. To consider these possibilities, we asked whether insect visitation rates differed among plant clusters associated with different landscape features. To estimate insect visitation, we summed pollinator visits to a cucumber cluster on each site‐date combination. Then, since each landscape feature had two plant clusters, we chose the lower visitation value from each pair as a conservative estimate of the insect abundance that would limit dye transfer across that feature. For landscape features that were adjacent to each other and had a shared cluster (Figure 1B), the shared cluster was considered in both estimates.

To assess the relationship between insect visitation and urban landscape features, we modeled this total visitation as a function of the landscape feature with a Poisson distribution and log link. We accounted for differences in sites, weather conditions, and cluster placements of observations by including random intercepts of cluster nested in group nested in date nested in site. Additionally, we included the log of the total cucumber flowers observed per cluster as an offset variable.

## 3 Results

### 3.1 Insect Pollinator Movement Across Urban Landscape Features

**FIGURE 2:**
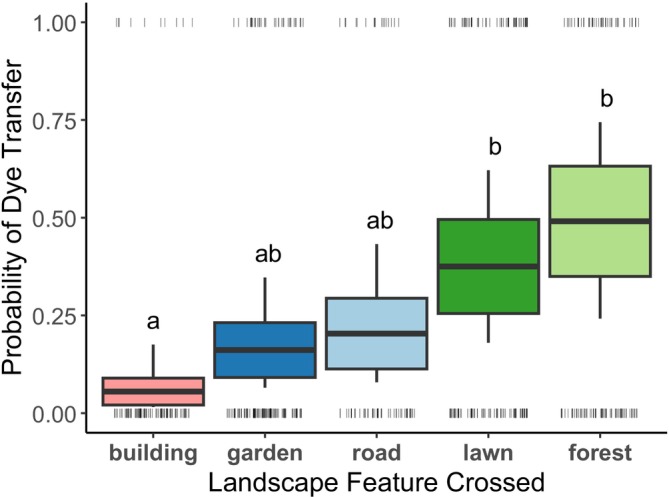
Buildings had the lowest probability to be crossed by urban pollinators (estimated probability = 0.055), while lawns (0.38), and suburban forest fragments (0.49) had the highest. Gardens (0.16) and roads (0.20) were intermediate. Features differed in the probability of crossing (*X* ^*2*^ = 15.9, df = 4, *p* = 0.003), and those sharing a common letter designation were not significantly different in a post hoc Tukey test. Boxes represent model‐predicted means ± SE, and whiskers are 95% confidence intervals. Each gray data point (tick mark) is one stigma: either 0 (dye absent) or 1 (dye present), jittered horizontally for visibility.

In total, we counted 5331 dye particles on 457 cucumber stigmas from 2 sampling days per site across 5 sites, testing 5 common urban landscape features. Probability of dye transfer differed between landscape features for both the dye particle absence/presence (*X* ^*2*^ = 15.9, df = 4, *p* = 0.003) and abundance (*X* ^*2*^ = 23.3, df = 4, *p* = 0.0001). This indicates that insects transferred dye differentially across landscape features. Based on the absence/presence data, post hoc comparisons indicated that buildings had the lowest probability of dye transfer (0.055, i.e., a flower had only a 5.5% chance of receiving dye from the other side of a building), while lawns and forest fragments had the highest probability (0.38 and 0.49, respectively) (Figure 2 and Table A3). For comparison, only 10 of 90 stigmas (11%) on building‐associated plants received dye from across a building, while 44 of 166 (26.5%) stigmas on forest‐associated plants received dye from across the forest fragment. Finally, gardens and roads had intermediate probabilities of dye transfer (0.16 and 0.20, respectively). Results were consistent when we analyzed dye particle counts instead of absence/presence (Table A4).

### 3.2 Insect Visitation to Cucumber Flowers

**FIGURE 3:**
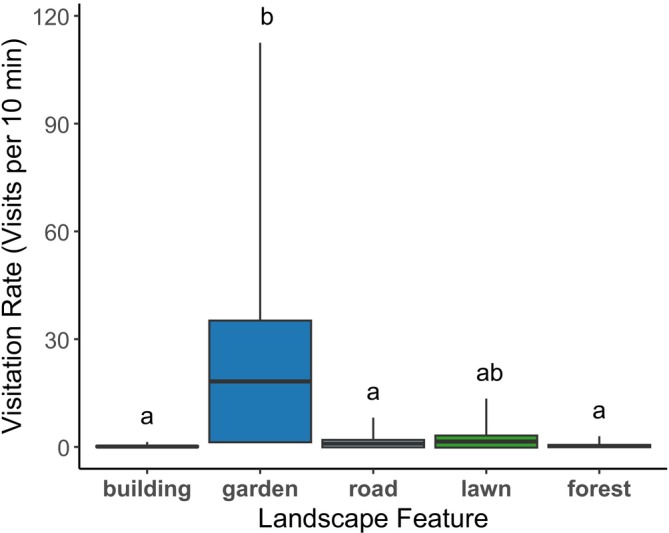
Insect visitation according to sampling month (June or July) and site, where each number (1–5) is a site. *Bombus* spp., *Apis mellifera* , and *Xylocopa viriginica* were the most common visitors, implying that our results are representative of large bees. The “other” visitors included *Halictus* spp., hoverflies (*Toxomerus*), skippers (Hesperiidae), and other flies (Diptera).

Overall, we observed a total of 769 pollinator visits during the timed observations of focal cucumber plants across all sites and dates. These included bees ( *Apis mellifera* Linnaeus, *Xylocopa virginica* [Linnaeus], *Bombus* spp., *Lasioglossum* spp., *Halictus* spp., and *Megachile* spp.), hoverflies (*Toxomerus*), skippers (Hesperiidae), and other flies (Diptera). Of these, *Bombus*, *Apis*, and *Xylocopa* were the most frequent visitors, together accounting for 88%–100% of all observed visits to cucumber flowers during timed observations on each site‐date combination (Figure 3 and Table A5). In other words, our dye transfer results primarily represent the activity and movement of large, generalist bees.

**FIGURE 4:**
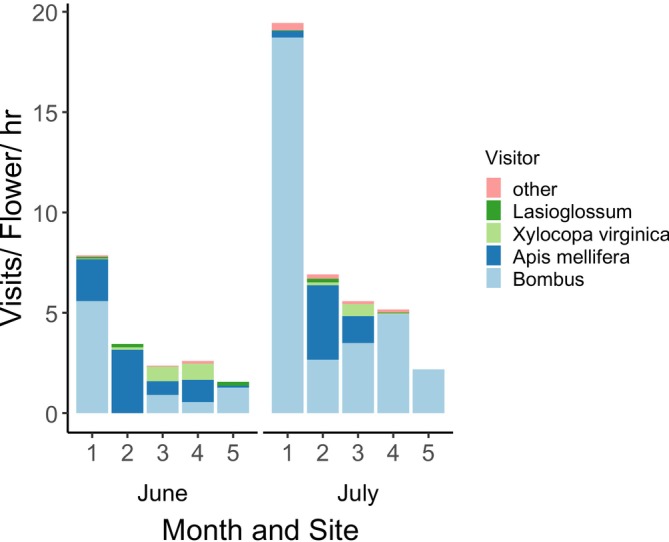
Cucumber clusters associated with gardens had the highest rate of pollinator visits per 10‐min observation period (mean 18.2 visits/10 min). Buildings (0.11 visits/10 min), roads (0.92 visits/10 min), and forest fragments (0.24 visits/10 min) had the lowest rates, while lawns were intermediate (1.5 visits/10 min). Visitation rate to clusters depended on landscape feature (*X* ^*2*^ = 23.4, df = 4, *p* = 0.0001); features sharing a common letter designation were not significantly different in a post hoc Tukey test. Boxes represent the model‐predicted mean rate of visitation ± SE, and the whiskers are 95% confidence intervals. The model included the number of flowers observed as an offset, and the estimated rate represents visits per cluster while holding the offset constant at its mean.

**FIGURE 5:**
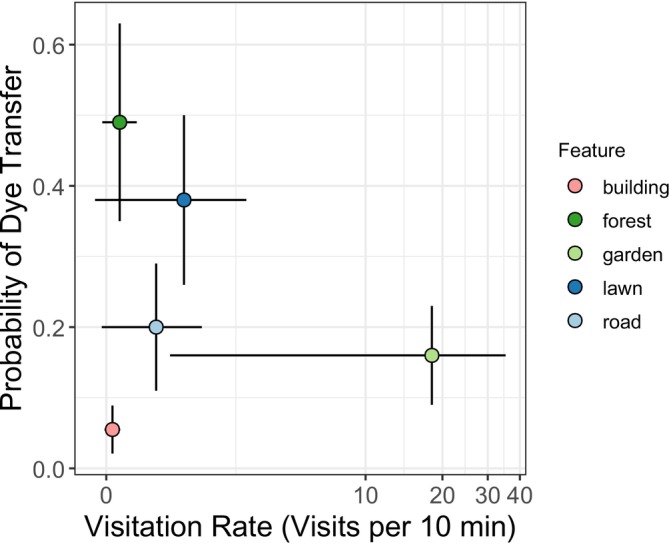
Visitation rate (from Figure 4) compared to probability of dye transfer (from Figure 2). Visitation rate was not a reliable predictor of dye transfer. Dots indicate each urban landscape feature type across all sites and dates, and lines represent ± SE.

The rate of insect visitation differed among plant clusters associated with different landscape features (*X* ^*2*^ = 23.4, df = 4, *p* = 0.0001). Clusters of cucumbers placed near gardens had the highest visitation rates (mean 18.2 visits/10 min) while those near buildings, forest fragments, and roads had the lowest rates (buildings 0.11, forests 0.24, and roads 0.92 visits/10 min, Figure 4 and Table A6). Lawns were intermediate at 1.5 visits/10 min. While buildings had both the lowest visitation and lowest dye transfer, visitation was overall a poor predictor of dye transfer (Figure 5). Namely, forest fragments had high dye transfer and low visitation, while gardens had low dye transfer and high visitation.

## 4 Discussion

Overall, our study shows that urban landscape features differ in how readily insect pollinators move pollen across them. This differential movement has important implications for urban pollinator conservation since pollinator movement determines the ease with which these insects may access resources and mediate plant pollen dispersal. These results also emphasize that Euclidean distance may be a poor indicator of how insects access resources and move pollen through heterogeneous city landscapes (see also Johansson et al. 2018). Although all of our cucumber clusters were separated by similar Euclidean distances (20–30 m), the rate of dye transfer (and implied pollinator movement) between them depended on the identity of the intervening landscape feature. Urban pollinator conservationists and researchers will likely need to consider these more complex effects of various landscape features on pollinator movement and resource availability; a bee may be less likely to access resources on both sides of a building, even if they are within its nominal flight range.

Moreover, our study also indicates that pollinator abundance in the vicinity of a landscape feature is not a good predictor of movement across that feature. For example, while cucumber flowers adjacent to forest fragments, buildings, and roads all had similarly low visitation rates, those separated by forest fragments were far more connected by dye transfer than were those separated by buildings. This pattern indicates that pollinators visiting cucumber flowers on one side of a forest fragment were likely to proceed directly to the cucumber flowers on the other side of the forest—whereas buildings effectively blocked such movement. Gardens also provided a striking contrast between visitation and dye transfer. Although visitation was more than 10 times greater for cucumber flowers adjacent to gardens than for any other landscape feature, dye transfer across gardens was low, second only to buildings. Here, we suspect that our visiting generalist pollinators took sinuous paths through foraging habitat in the gardens (e.g., Cresswell 2007; Kuefler et al. 2010), and either failed to reach the other side entirely or depleted the dye from their bodies before they did so. Although many urban pollinator studies survey pollinator communities associated with different urban habitat types, our results emphasize that abundance or visitation rates are not proxies for pollen or pollinator connectivity.

Of all the urban landscape features studied, buildings had the lowest estimated dye transfer probability—significantly lower than lawns and forests—suggesting that buildings are a barrier to insect pollinators. Our movement result supports the expert opinion of Johansson et al. (2018), who assigned buildings with 10 times higher “friction” than any other urban landscape feature (where high friction represents low probability of movement). The mechanisms by which buildings affect pollinator movement are not known. They may simply pose a visual barrier, but they might also contribute to patches of high temperatures that some pollinators are sensitive to (Hamblin et al. 2017; Hong et al. 2021). Additionally, buildings often produce polarized light pollution which may affect how certain insects navigate (Horváth et al. 2009; Kraft et al. 2011), potentially reducing how effectively pollinators can move when near these structures. Moreover, many central‐place pollinators, like the *Bombus* spp. and *Apis mellifera* encountered in our study, use linear landmarks—such as roads—to navigate routes (Brebner et al. 2021; Bullinger et al. 2023). Whether the linear structure of buildings help or hinder this process has yet to be explored and may be another focus for future research.

Roads had the next lowest estimated dye transfer probability, which agrees with other studies demonstrating the restrictive effect of roads on pollinator movement (Askling and Bergman 2003; Dániel‐Ferreira et al. 2022; Fitch and Vaidya 2021; Markovits et al. 2025). Given that road characteristics, such as size, traffic intensity, and floral resources on roadsides can affect road resistance to pollinators (Dániel‐Ferreira et al. 2022; Fitch and Vaidya 2021; Keilsohn et al. 2018; Martin et al. 2018), our result—based on two‐lane, 45 mph roads—is likely a conservative estimate of how roads alter insect pollinator movement.

Lawns had one of the highest estimated dye transfer probabilities, indicating that this feature may facilitate insect pollinator movement in urban spaces. Likely, these results are because lawns are easy to see and travel across to different resource patches. The extent to which urban lawns aid pollinators likely depends on how heavily managed the lawns are (Proske et al. 2022). The lawns in this study were periodically mowed, often with patches of wildflowers such as dandelions and clovers that might facilitate movement between patches or better attract/support an urban bee community.

Finally, forest fragments had the highest estimated dye transfer probability, suggesting that forest fragments may also facilitate insect pollinator movement. Although we expected forests to function as visual barriers, similar to buildings, our results instead imply that cucumbers separated by forest fragments were visited by few, highly effective pollinators. The observed visitor composition to forest‐associated clusters was similar to those at all other clusters and was dominated by bumble bees. Our observations may have missed other visitor taxa unique to the forest‐associated clusters, or bumble bees transiting forests may have been particularly likely to visit flowers on both sides of the forest fragments, thus connecting them via dye transfer. Mola et al. (2020) also found no inhibitory effect of forests on bumble bee foraging movements in an alpine setting, but additional research is needed to identify which species or behaviors mediated the high rate of dye transfer by relatively few individual visitors in our study.

Of the insect pollinators who visited our cucumber clusters, we mainly saw large generalist bees: *Apis mellifera* , *Bombus* spp., and *Xylocopa virginica* . This implies that much of the dye was transferred by these species. Since large bees, most notably *Bombus* spp., are able to deposit more pollen (and presumably dye) per visit (e.g., Cariveau et al. 2013), movements of small species are undoubtedly under‐represented in our dataset. As large bees and social bees tend to be the most mobile species (Kendall et al. 2022), and may be less affected by urban barriers (Fitch and Vaidya 2021), continued research into urban landscape connectivity for small and solitary bees is particularly needed. Specialist pollinators may also interact differently with the landscape as they seek a limited subset of available floral resources (e.g., Zurbuchen et al. 2010). In general, more research into physiological and behavioral characteristics (i.e., size, specialist vs. generalist, eusocial vs. solitary) of insect pollinators in the context of urban movement is needed, as our study is primarily focused on central‐place, generalist foragers.

Overall, this study provided a broad overview of the effects of different common urban landscape features on bee and pollen movement. Now that we have quantified the strong and contrasting effects of certain features—particularly buildings and forests—on the movement of a generalist pollinator community, there is a need for additional research to uncover the mechanisms and behaviors underlying these effects. For example, future studies could address a gradient of building dimensions, forest depth/thickness, or interactions between features and floral or nesting resources. Future studies may also use not only dye transfer but methods that directly detect movement such as mark‐recapture or telemetry. Additionally, studying the interactions between landscape features could reveal important management implications [e.g., the effects of roadside lawn and meadow habitat on insect mortality (Keilsohn et al. 2018]). We recommend that cities focus future conservation and management on how and where pollinators move in the urban landscape to create networks of pathways between pollinator habitats, suitable for insect and pollen dispersal through the city (Townsend and Levey 2005; Van Rossum 2010). For example, our results can be used to configure resistance maps and to estimate habitat connectivity between resource patches for pollinators in a city (e.g., Unnithan Kumar et al. 2022). With more quantitative movement data of urban insect pollinators, conservation practitioners can be better informed on how and where to implement such networks.

## Author Contributions

**Olivér I. Roper:** conceptualization (equal), data curation (lead), formal analysis (lead), funding acquisition (lead), investigation (lead), methodology (equal), project administration (lead), visualization (lead), writing – original draft (lead), writing – review and editing (equal). **Elsa Youngsteadt:** conceptualization (equal), formal analysis (supporting), investigation (supporting), methodology (equal), resources (lead), visualization (supporting), writing – review and editing (lead).

## Conflicts of Interest

The authors declare no conflicts of interest.

## Data Availability

The data that support the findings of this study are openly available in Dryad at https://doi.org/10.5061/dryad.w9ghx3g08.

## Appendix A

**TABLE A1:** Distances, means, and SDs between plant clusters per landscape feature in m.

	Site 1	Site 2	Site 3	Site 4	Site 5	Mean	SD
Building	26	27	27	21	NA	25	3
Garden	20	23	26	20	22	22	3
Forest fragment	25	20	NA	26	28	24	4
Lawn	20	NA	23	27	23	23	3
Road	30	24	20	25	NA	25	4

*Note:* Distances between plant clusters were 20–30 m and were measured to ensure that differences in distance did not bias our results. Here, we report the distance between clusters for each feature at each site, as well as the overall mean and SD across sites for each feature type. The distances between clusters did vary, but this variation was independent of the feature that separated them (*F*_4,16_ = 0.73, *p* = 0.58).

**TABLE A2:** Bee specimens collected from cucumber flowers during an ongoing pollinator study at our five community garden sites.

Family	Species	*n* specimens
Halictidae	*Agapostemon sericeus*	1
Halictidae	*Augochlora pura*	1
Halictidae	*Lasioglossum callidum*	1
Apidae	*Apis mellifera*	23
Apidae	*Bombus griseocollis*	1
Apidae	*Bombus impatiens*	75
Apidae	*Ceratina calcarata*	3
Apidae	*Xylocopa virginica*	2
Megachilidae	*Megachile campanulae*	1
Megachilidae	*Megachile mendica*	1

*Note:* Prior to the current study, we collected a total of 109 bees from cucumber flowers during timed collections at the same sites in June 2021, July 2021, June 2022, and July 2022. Each site was sampled 4 times with a total sampling effort of 48 min per site, distributed across all flowering plants growing at a site. The specimens collected from cucumber—listed here—provide the basis of our field identifications in our current study. Other pollinator taxa (e.g., butterflies and flies) were not collected.

**TABLE A3:** Fixed‐effect estimates from generalized linear mixed model of the presence/absence (1/0) of dye particles transferred as a function of landscape feature.

	Probability (of dye transfer between clusters)	SE	Group
Building	0.055	0.034	a
Garden	0.16	0.07	ab
Road	0.20	0.09	ab
Lawn	0.38	0.12	b
Forest fragment	0.49	0.14	b

*Note:* The model was significant (*X* ^*2*^ = 15.9, df = 4, *p* = 0.003), and groups that share a common letter designation were not significantly different in Tukey pairwise comparisons (i.e., a and b are significantly different from each other but not from ab).Thus, insects were significantly less likely to move across a building than across a lawn or forest.

**TABLE A4:** Fixed‐effect estimates from generalized linear mixed model of the number of dye particles transferred as a function of landscape feature.

	Rate (of dye transfer between clusters)	SE	Group
Building	0.0043	0.0038	a
Garden	0.032	0.025	ab
Road	0.093	0.083	ab
Lawn	0.19	0.15	b
Forest fragment	0.48	0.43	b

*Note:* Results were consistent with the analysis based on dye absence/presence presented in the main text, Figure 2, and Table A3 in that the same landscape features statistically differed from, or were similar to, each other. The full model was significant (*X* ^*2*^ = 23.3, df = 4, *p* = 0.0001); groups sharing a common letter designation were not significantly different in Tukey pairwise comparisons.

**TABLE A5:** Percent of visits by large bees during 10‐min observation periods at each cucumber cluster per site and round.

Site	Round	*Bombus* spp.	*Apis mellifera*	*Xylocopa virginica*	*Lasioglossum* spp.	Other	Total flowers	Total visits	Total large	% Large
1	June	147	55	1	2	2	158	207	203	98
2	June	0	78	3	4	0	148	85	81	95
3	June	20	15	16	0	1	132	52	51	98
4	June	13	26	19	0	3	141	61	58	95
5	June	27	2	0	4	0	127	33	29	88
1	July	624	11	0	1	12	200	648	635	98
2	July	114	159	6	8	9	257	296	279	94
3	July	99	38	17	0	4	170	158	154	97
4	July	130	0	1	1	4	158	136	131	96
5	July	56	0	0	0	0	154	56	56	100

*Note:* Individuals were identified on the wing so some bees were identified only to genus; see Table A2 for the species composition of these genera in specimen‐based collections from the same sites. The ‘Other’ category includes taxonomic groups that were comparatively uncommon: *Ceratina* (Hymenoptera: Apidae), *Halictus* (Hymenoptera: Halictidae), *Agapostemon* (Hymenoptera: Halictidae), *Megachile* (Hymenoptera: Megachilidae), *Polistes* (Hymenoptera: Vespidae), *Vespula* (Hymenoptera: Vespidae), Hesperiidae (Lepidoptera), *Cotinis nitida* (Coleoptera: Scarabaeidae), *Toxomerus* (Diptera: Syrphidae), and other Diptera. Total flowers was the sum of all flowers that were observed at each site/round, total visits was the sum of all insect visitors per site/round, and total large was the sum of *Bombus* spp., *Apis mellifera* , and *Xylocopa virginica* visits at each site/round. Percent of large bees was calculated as the total large bee visits out of the total insect visits, and represented 88%–100% of visitors across sites/rounds.

**TABLE A6:** Insect visitation estimated from a generalized linear mixed model of total insect visits as a function of landscape feature.

	Rate (insect visits per 10 min)	SE	Group
Building	0.11	0.14	a
Garden	18.2	17	b
Road	0.92	1	a
Lawn	1.5	1.7	ab
Forest fragment	0.24	0.31	a

*Note:* Observations were 10 min per cluster, and the lowest number of total visits out of a group was used in the model as a conservative indicator of the availability of insects that could potentially move between clusters. The model was significant (*X* ^*2*^ = 23.4, df = 4, *p* = 0.0001), and groups that share common letter designation were not significantly different in Tukey pairwise comparisons. Thus, plants adjacent to different landscape features statistically differed in the frequency of insect visitation.
